# Clinical Uses of Ketamine in Children: A Narrative Review

**DOI:** 10.7759/cureus.27065

**Published:** 2022-07-20

**Authors:** Anoushka Bali, Ashujot Kaur Dang, Daniel A Gonzalez, Rajeswar Kumar, Saba Asif

**Affiliations:** 1 Research, Acharya Shri Chander College of Medical Sciences & Hospital, Jammu, IND; 2 Research, Government Medical College Patiala, Patiala, IND; 3 General Medicine, Universidad Catolica Santiago de Guayaquil, Guayaquil, ECU; 4 Medicine, Rajah Muthiah Medical College and Hospital, Chidambaram, IND; 5 Internal Medicine, Apollo Hospitals, Hyderabad, IND

**Keywords:** pediatric population, clinical uses, anesthesia, sedative, ketamine

## Abstract

Ketamine is a phencyclidine derivative that acts as a noncompetitive N-methyl-D-aspartate as well as a glutamate receptor antagonist. It also has other minor mechanisms that contribute to its extensive drug profile. Ketamine is a bronchodilator and maintains normal airway reflexes and, thus, permits spontaneous respiration. This, coupled with the fact that it produces potent analgesia, makes it highly suitable for children.

Despite its many merits, the drug’s side effects, along with its cultural image of being a drug of abuse, a drug used in veterinary medicine, or a “date-rape drug” have sullied its reputation within the armamentarium of medicine. Even though it is widely used in developing countries, its use in Western nations has diminished. We have strived to explore the various clinical uses of ketamine in children through this article. In addition, the article also highlights how some of the fears associated with using the drug are unfounded and provides ways by which the drug’s side effects can be prevented and managed.

## Introduction and background

Ketamine has been used extensively as an intravenous (IV) anesthetic agent for the past 65 years [1]. When given as IV, ketamine produces dissociative anesthesia, a trance-like state characterized by catatonia, catalepsy, analgesia, and amnesia. The patient’s eyes remain open with intact corneal and light reflexes during the procedure. The drug also keeps the pharyngeal and laryngeal reflexes preserved, due to which the risk of airway obstruction is minimum. There is a feeling of being detached from the surroundings, with distortion of visual and auditory stimuli [2]. Ketamine has a unique pharmacological profile due to its action on multiple molecular targets in the central nervous system (CNS). Its antagonism at the N-methyl-D-aspartate (NMDA) receptors is largely responsible for dissociative anesthesia and its potential neuroprotective effect [3]. However, the drug’s unique and varied pharmacological profile cannot be explained with this mechanism alone. Its action on the opiate receptors produces rapid and profound analgesia, while its action on monoaminergic, cholinergic, nicotinic, and muscarinic receptors is responsible for its hypnotic and psychic effects [1]. Peripherally, ketamine has a sympathomimetic action and increases blood pressure (BP) and heart rate (HR), a quality that is extremely valuable in the emergency setting for patients who are hypotensive or in shock [4]. It can be administered via almost any route, including IV, intramuscular (IM), oral, and intranasal, although the first two are the most popular [2].

Ketamine has a distinctive role for the pediatric population and has several advantages over other anesthetics. It is the drug of choice for children with congenital heart diseases (CHD) with a right to left shunt because of its beneficial cardiovascular effects [5]. It is used extensively in head and neck surgeries, including dental procedures and cleft palate repair operations [5]. Since ketamine preserves the airway reflexes and is a bronchodilator, it is extremely useful for anesthetizing patients with reactive airway disease [6]. In addition, the drug has several non-anesthetic uses in clinical practice. Ketamine can be used as an analgesic for managing postoperative pain. It has also proven to be an excellent drug for the treatment of chronic pain. It is used as a rapid sedative-analgesic to aid in short and painful procedures in the emergency department [5]. It is also used for burns dressings and wound care [7]. Newer research has concluded that ketamine has efficacy in managing treatment-resistant depression, bipolar depression, and post-traumatic stress disorder (PTSD) [8].

Despite being a drug with excellent safety and efficacy, ketamine suffers from a deep stigma in society for being a drug used in veterinary medicine and for having abuse potential, which has limited its use in developed countries [5]. Many physicians continue to be wary of using the drug in clinical practice due to misinformation about its risks and complications. Many newer studies have suggested that most of these fears are largely unfounded [9]. On the other hand, the drug’s abuse potential and its reinforcing effects have made it challenging to use it to treat psychiatric disorders [10].

This article aims to highlight the many uses of ketamine in the pediatric population while dispelling the false risks that are often associated with the drug. It also aims to shed light on the challenges that currently limit the usage of the drug and suggest ways in which clinicians can overcome them.

## Review

Brief history of ketamine

Ketamine was synthesized from phencyclidine to overcome the parent drug’s hallucinogenic side effects and abuse potential. Its first use in humans was on August 3, 1964, when it was administered to volunteer prisoners at the Jackson Prison in Michigan, United States, by Domino et al. [11]. Initially, it only produced an out-of-body experience in the subject, but on increasing the dose, a state of general anesthesia was produced [1]. The volunteers described the experience as “floating in outer space with no sensations in their arms and legs.” This was termed “dissociative anesthesia,” a term that is still used to describe the effects of ketamine. Dissociative anesthesia was later described as an electrophysiological and functional disconnect (or “dissociation”) between the thalamocortical and limbic areas of the brain [12]. Parke-Davies patented ketamine for human and animal use in 1966. In 1969, it became available on prescription as ketamine hydrochloride, with the United States Food and Drug Administration (FDA) officially approving it for human use in 1970 [1].

When it was first introduced, ketamine was deemed an ideal and complete anesthetic since it fulfilled all the requirements for surgical anesthesia, i.e., blocked sensory, motor, autonomic, and cognitive functions [13]. However, just like its parent molecule, ketamine too produced an emergence phenomenon in the postoperative period, a state in which the patient experiences vivid dreams and could become extremely irritable and agitated. This was found to be more common in adults than children. This side effect led to a decline in the drug’s popularity among anesthesiologists [9].

However, a study of prospective data collection records conducted by Treston et al. suggested that this emergence delirium occurs in only 2.1% of children, with 39% experiencing pleasant altered perceptions. Also, none of the children in the study required any active treatment for it, and none experienced any long-term effects of this experience in the form of nightmares [14].

Pharmacokinetics

Administration

Ketamine is soluble in water as well as lipids. Thus, it can be given via almost any route, including IV, IM, subcutaneous, inhaled, oral, intrarectal, or epidural, with the first two being the most popular [1]. Recently, intranasal and sublingual formulations of ketamine have been developed, with the former providing rapid absorption and high bioavailability. Rectal administration of ketamine is a good alternative to the IV route for obtaining analgesia in children in cases where an IV route cannot be established [3,15].

Absorption

The drug has a transfer half-life of less than a minute with the IV route. The bioavailability is 90% by IM route, 77% by epidural, 50% by intranasal, 25% by intrarectal, and 20% by oral route (due to first-pass metabolism in the liver). IM absorption is faster in children due to lower muscle mass and higher perfusion at the site [3,16].

Distribution

Ketamine is highly lipid-soluble and has only 20-50% protein binding, showing more affinity for α1- acid glycoprotein than albumin. As a result, it has a very large volume of distribution (3-5 L/kg). It crosses the blood-brain barrier rapidly to induce anesthesia. Redistribution of the drug from the brain to the other tissues and plasma is responsible for the termination of anesthesia [1].

Metabolism

Ketamine undergoes extensive metabolism by the hepatic microsomal enzymes. It is demethylated to form norketamine by CYP3A4. Norketamine is the major metabolite and has some anesthetic effect, along with the psychoactive properties of ketamine. Animal studies have suggested that it crosses the blood-brain barrier and has one-fifth to one-third the potency of ketamine [17]. Studies suggest that the activity of CYP3A4 is greater in infants and children than in adults, the most dramatic differences being during the first six months of life. This difference can be a major influence on the rates of metabolism seen in children versus the ones seen in adults [18]. Ketamine also exhibits self-induction by inducing the enzymes responsible for its metabolism. This can lead to the development of drug tolerance with chronic administration. Ketamine and norketamine are hydroxylated to form glucuronide derivatives that are highly water-soluble and easily excreted [3].

Elimination

The plasma clearance in children is around 16.8 ml/kg/min. The elimination half-life is shorter (100 minutes) than that of adults. In the first three months of life, however, the plasma clearance is shorter due to decreased metabolic capacity of the liver. The median value for the elimination half-life of per-rectal ketamine (3.15 h) is considerably longer than that reported after IV and IM administration of the drug (0.8-1 h) to pediatric patients. In urine, 80% of ketamine is excreted as glucuronic acid conjugates, 16% as dehydronorketamine, and 2% in the unchanged form [1].

Malinovsky et al. conducted a study to understand the pharmacokinetics of ketamine in children. Thirty-two children aged 2-9 years and falling in the weight range of 10-30 kg who were undergoing a minor urological surgery were selected [16]. They were divided into groups and administered ketamine via three different routes: IV, intranasal, and intrarectal. The first group received 3 mg/kg ketamine IV, the second received 3 mg/kg ketamine intranasally, and the third received 9 mg/kg ketamine intrarectally through a short rectal cannula. The plasma values of ketamine and norketamine were measured using gas-liquid chromatography. The peak plasma concentration with intranasal administration appeared after 20 minutes, while it appeared after 42 minutes with intrarectal administration. It was found that the calculated bioavailability was 0.50 with intranasal administration and 0.25 with intrarectal administration [16].

Clinical applications in the pediatric population

Ketamine holds a unique and invaluable position in pediatric anesthesia, having regained its popularity in the prehospital, emergency department, and operation room setting in the last decade. Its advantages as an anesthetic lie in the fact that it preserves respiratory drive and does not lead to hypotension.

Anesthetic Uses

Ketamine is used for the induction and maintenance of anesthesia for various operations. It remains a mainstay of anesthesia in several developing countries due to its rapid induction and safety profile. Even though some newer anesthetics have replaced its use nowadays, it remains the drug of choice in pediatric anesthesia for children with a difficult airway, reactive airway disease, and uncooperative children who require IV access [6].

Ketamine is an ideal prehospital anesthetic agent since it produces rapid and predictable anesthesia while maintaining hemodynamic stability and respiratory reflexes. Additionally, the drug has a versatile therapeutic profile, produces potent analgesia and sedation, and has a good safety profile. It has the distinct advantage of providing cardiovascular stability due to its sympathomimetic effect, which compensates for its direct negative inotropic action on an isolated heart. It increases the HR, BP, and cardiac output but relaxes bronchial smooth muscles [4]. This makes the drug effective for patients in shock, hypotensive patients, and ones with respiratory or cardiac problems. A randomized control trial by Jabre et al. claimed that it can be used as an effective and safe alternative to etomidate for rapid endotracheal intubation in acutely ill patients and should be considered for sepsis [19]. Ketamine is the only intravenous anesthetic that increases the mean arterial pressure without compromising the cardiac output. In addition, experimental and clinical studies have shown that it has anti-inflammatory properties and inhibits the release of proinflammatory cytokines like interleukin-6 and tumor necrosis factor-alpha [20].

There is also evidence that ketamine can be used for head trauma because of its neuroprotective effects against cerebral ischemia [21]. Prospective studies pertaining to 55 pediatric patients concluded that contrary to popular belief that ketamine increases the intracranial pressure (ICP), a bolus dose of the drug actually significantly decreased the ICP while increasing the cerebral perfusion and mean arterial pressure [22]. However, Hill et al. deduced that there was no difference in mortality between pediatric patients who were administered ketamine for head trauma in the prehospital setting from those who were not administered the drug [23]. Ketamine is considered useful for patients with cardiac tamponade due to trauma since the drug does not have a deleterious effect on afterload and maintains perfusion to the vital organs [24,25]. In addition, the drug shows its effect within minutes and can be used to restrain and anesthetize uncooperative patients, including children with intellectual deficits [5,26].

Ketamine is widely used as a field anesthetic, especially in under-resourced areas. The United States army used it during the Vietnam war [27]. During the 2005 earthquake in Northern Pakistan, a total of 149 patients received emergency surgery in Kashmir using ketamine anesthesia with benzodiazepine premedication [28]. Due to the absence of anesthesia machines and ventilators during the 2010 earthquake in Haiti, ketamine was utilized as a general anesthetic [29]. It was also deemed the anesthetic of choice by the 23-member Australian medical team in Banda Aceh, Indonesia, for the victims of the 2004 Boxing Day tsunami [30]. Ketamine was used on 164 awake non-trapped pediatric patients with blunt trauma by London Hospital medical service and was found to be highly efficacious and safe [31]. Since it also minimizes the decrease in core temperature, which usually occurs with anesthesia, it could theoretically benefit hypothermic patients. But this particular use has not been extensively studied [32].

In the operating room, it is used as a premedication, regional anesthetic, general anesthetic, and adjunct to other general anesthetics. Ketamine is used for induction in children because of its rapid onset of action. Different routes can be used for this, including IV, IM, oral, and intranasal. It allays preoperative anxiety in children and improves their cooperation during procedures [33]. A study by Cossovel et al. suggested that the combination of intranasal dexmedetomidine in the dose of 2μg/kg and oral ketamine in the dose of 3mg/kg can help in satisfactory separation of the child from the caretaker, allow better success rates of venous cannulation, and smooth induction of general anesthesia [34]. According to Darlong et al., the combination of 3 mg/kg oral ketamine and 0.25 mg/kg midazolam has minimal side effects and allows rapid induction of anesthesia when compared to using either of the drugs alone [35].

Ketamine in the concentration of 0.5 and 0.3% produces adequate intravenous regional anesthesia. Caudally administered ketamine can be combined with a local anesthetic to provide prolonged postoperative analgesia and fewer side effects relative to using the local anesthetic alone [36]. In inguinal herniotomy procedures, ketamine added to bupivacaine provided longer analgesia than when the latter was used alone. Cook et al. demonstrated that the combination of ketamine in the dose of 0.5 mg/kg with bupivacaine provided longer-lasting postoperative anesthesia after orchidopexy than the combination of clonidine in the dose of 2μg/kg or epinephrine in the dose of 5μg/kg with bupivacaine [37]. Hence, caudal blocks under basal ketamine are widely used for abdominal and lower limb surgeries, especially for uncooperative children. Intravenous ketamine in the dose of 0.5 mg/kg can be given prophylactically before neuraxial block to decrease the incidence of shivering, improve analgesia, and prevent the recall of the surgical events [38]. It can also be combined with regional anesthetics for ocular surgeries in children [39].

Ketamine is the general anesthetic of choice for children with congenital heart diseases (CHD) with a right to left shunt, like the tetralogy of Fallot, because of its beneficial cardiovascular effects [40]. It increases the systemic vascular resistance, decreasing the venous return and, hence, decreases the shunting of blood in the heart [41]. It is used widely for head and neck surgeries like tonsillectomies, cleft palate repair operations, and dental procedures [42-44]. Studies suggest that it can be used in surgeries for traumatic brain injuries with increased intracranial pressure (ICP) since it decreases ICP without decreasing the systemic BP and cerebral perfusion [22]. It is also the agent of choice for induction in children with reactive airway disease and difficult airways because of its broncho-dilating effect [6]. When ketamine is used as a co-induction agent with propofol, midazolam, or dexmedetomidine, it provides the benefits of hemodynamic stability, reduced pain on injection, and less respiration depression [34,35].

Non-Anesthetic Uses

Ketamine has several uses outside of its value as an anesthetic agent. These uses are attributed to its interaction with NMDA, opioid, cholinergic, monoaminergic, purinergic, and adrenergic receptors [1].

Its analgesic effect is due to its action on opioid receptors as well as its propensity to decrease nociceptive stimuli [5]. Ketamine is an ideal drug for prehospital analgesia since it provides potent pain relief without jeopardizing the speaking capacity of the patient. It can be used as an analgesic for amputations, burns, and fracture reduction in the field. A combination of 40 mg of IV ketamine with 5 mg of IV morphine has proven to be more beneficial than using the latter alone. It can also be used as an analgesic while extracting a trapped patient in the case of disasters [45].

The sedative-hypnotic effect of ketamine is due to the antagonism of NMDA receptors and hyperpolarization-activated cation channels. Thus, the combination of ketamine and propofol mixed in the same syringe (called “ketofol”) is used widely for procedural sedation in infants and children [46]. This combination is advantageous because ketamine prevents hypotension associated with propofol, while propofol mitigates agitation and nausea associated with ketamine. It can be used for short and painful procedures like cardiac catheterization, radio studies, dressing changes, foreign body removal, laceration repair, fracture reduction, abscess drainage, emergency cardioversion, amputations, and chest tube insertion, especially in children who are uncooperative or have intellectual deficits [47-50]. It can also be used for long-term sedation in children with retinoblastoma [51]. Ketamine is used as a sedative-analgesic for attacks of severe acute respiratory distress syndrome (ARDS). It can also be used for sedation during hyper-cyanotic spells in children with tetralogy of Fallot [52].

Newer case reports and systemic reviews have illustrated that ketamine is a wonder drug for acute and chronic pain. Its action on NMDA receptors and its anti-inflammatory effect make it an excellent drug for postoperative pain relief. When combined with other drugs like propofol, it prolongs the postoperative analgesia. Intranasal ketamine can be used to provide postoperative analgesia after endoscopic nasal surgery [53]. Ketamine spray can be used in the tonsillar fossa to numb the area after a tonsillectomy [54]. Clinical trials have suggested that ketamine can be combined with opioids to reduce pain sores. This ketamine-opioid combination reduces the amount of morphine required to maintain the same level of analgesia [55]. It is also efficacious for postoperative pain relief after surgery for scoliosis in adolescents, as shown by a randomized control trial conducted by Minoshima et al. on 36 patients aged 10 to 19 years [56]. It is also used to manage the pain crisis in sickle cell disease patients [57].

Ketamine can also be utilized for chronic pain disorders, especially ones with a neuropathic component, like ischemic limb pain, phantom limb, neuropathic pain, fibromyalgia, complex regional pain syndrome (CRPS) type I, irritable bowel syndrome, and even migraine. An IV infusion of ketamine in the dose of 0.1 to 0.3 mg/kg/hour given over four to eight hours per day for 16 hours for three consecutive days has shown to significantly decrease the intensity of pain in adolescents and children with chronic pain, with the most benefit being seen in CRPS [58]. However, its use as an IV infusion or topical cream is mostly restricted to treatment-resistant neuropathic pain due to the lack of research. The drug has also shown efficacy in cancer pain, especially when used as an adjunct to opioids. Several randomized control trials have suggested the benefit of ketamine in opioid refractory cancer pain [59]. The drug is now on the World Health Organization (WHO) list of essential drugs for patients resistant to opioids or who have predictable breakthrough pains. Additionally, its NMDA blocking mechanism is theorized to have an anti-tumor effect [60].

Ketamine provides pain relief during dressing, excision, and grafting for burns and is safe and well-tolerated in children. It can be given by IM route in patients with severe burns in whom establishing a venous line is difficult [61,62].

New studies have shown that ketamine, particularly its esketamine (S-ketamine) enantiomer, is efficacious in major depressive disorder, post-traumatic stress disorder (PTSD), anxiety disorders, and bipolar depression [8]. A trial concluded that IV infusion of ketamine had higher remission rates and clinical response compared to a placebo [63]. It significantly improved the depression score in 127 cases of major depression in another trial [64]. A double-blinded clinical trial was conducted by Dwyer et al. to check the efficacy of ketamine on adolescents in the age group of 13 to 17 years who were diagnosed with major depressive disorder. The participants had previously tried at least one antidepressant drug and had a score of >40 on the Children’s Depression Rating Scale-Revised. The trial found that ketamine was well tolerated by the patients and showed significant short-term efficacy lasting for two weeks in comparison to a placebo [65].

A retrospective review has shown that the use of long-term oral ketamine in patients with PTSD reduced hospital admissions by 65% and in-patient hospital stays by 70% [8,66]. A double-blinded trial with 12 patients having treatment-resistant generalized anxiety disorder and social anxiety disorder elucidated that the administration of ketamine improved the anxiety rating of the participants within an hour, with the effects lasting for up to one week [67, 68]. A study also showed that using S-ketamine during electroconvulsive therapy reduced the number of sessions needed, produced lower depression scores, and improved cognitive ratings [69]. It has also been claimed that ketamine could be used for autism spectrum disorders. A 2006 case report concluded that ketamine produced a substantial but short-lived remission of the core symptoms of autism in a patient with severe intellectual disability [70].

Ketamine has a promising role in refractory status epilepticus, which occurs in 30% of cases of status epilepticus. Ketamine is used in the mean dose of 40 μg/kg/min for children and has proved to be quite effective in terminating the seizure [71].

Some lesser-known uses of ketamine include its use for treating postoperative sore throat when administered in the form of a gargle and its use in acute porphyria [72,73]. Although, the latter is considered controversial.

Procedure for anesthesia with ketamine

Preoperative Preparation

The details of the surgical procedure and its complications must be explained to the parent or caretaker, and informed consent must be obtained from them. The child’s baseline vitals, including HR, BP, respiratory rate (RR), temperature, and oxygen saturation must be recorded. The risk of unpleasant postoperative complications can be minimized by building a good rapport with the child. Some distraction techniques like toys or music can be used to take the child’s mind off the procedure [74]. If ketamine is supposed to be applied topically, it should be done at least 45 minutes prior to the operation since the drug takes time to get absorbed through the skin [75].

Administration

The drug route is selected based on the procedure and on whether IV access is available or not. IV route is the most widely used since it allows for the quickest recovery. An initial dose of 1 to 1.5 mg/kg is administered over one to two minutes before the procedure. Subsequent doses of 0.25-0.5 mg/kg can be given every 10 minutes (maximum 4.5 mg/kg) if they are required. However, doses more than 2.5 mg/kg are associated with adverse events like hemodynamic instability. Hence, if higher doses are required, the anesthesiologist must consider alternatives to ketamine like midazolam and propofol [76].

The IM route may be used if IV access cannot be obtained, for example in a severely dehydrated child. An initial dose of 4 mg/kg (maximum being 6 mg/kg) is given, followed by doses of 2mg/kg repeated every 10 minutes (if required). The IM route is handy in remote areas or emergencies where sophisticated equipment is not available [77].

Oral ketamine can be used as a pre-anesthetic medication. The taste of the oral solution of ketamine in children can be masked by using sour cherry juice or cola-flavored drinks. A study by Gutstein et al. has suggested that administering oral ketamine with flavored fluids before induction of general anesthesia does not increase the risk of aspiration, and is thus safe for children who refuse to accept oral ketamine because of its unpalatable taste [33]. Orally, ketamine is generally given in the dose of 6 mg/kg. This dose provides adequate and predictable sedation. It allows calm separation of the child from the parent or caretaker and is associated with minimal side effects. Table 1 discusses the routes of administration of ketamine. 

**Table 1:** Routes of administration of ketamine

Study	Route of administration	Time taken for onset of anesthesia	Time taken for termination of anesthesia	Dose
Dallimore et al., 2008 [76]	Intravenous	1-2 minutes	20-60 minutes	1 to 1.5 mg/kg is administered over 1-2 minutes followed by 0.25-0.5 mg/kg every 10 minutes
Green et al., 1998 [77]	Intramuscular	10-15 minutes	30-120 minutes	4 mg/kg, followed by doses of 2mg/kg repeated every 10 minutes (if required).
Gutstein et al., 1992 [33]	Oral	20-30 minutes	60-90 minutes	6 mg/kg

Postoperative Care

The child should be monitored at the hospital till they return to their usual state of health. They should be kept in a quiet and dimly lit environment to avoid agitation. The parents or caretaker should be alerted that the child may feel nauseated or vomit on their way home.

Side Effects and Their Management

Despite its excellent safety profile, the drug has some well-known side effects and limitations. It is known to produce tachycardia and hypotension. These are linked to the sympathomimetic action of ketamine and are usually transient [78]. The drug is contraindicated in infants younger than three months due to its propensity to produce laryngospasm [79]. Another reason why the drug is considered unsafe for neonates is because of the risk of it decreasing the respiratory drive and causing apnea. Its use in infants up to 12 months old is also considered controversial. Some physicians prefer to not use ketamine in children suffering from upper respiratory tract infections because of the risk of laryngospasm. However, there is data that proves that these concerns are somewhat unfounded. Green et al. analyzed a sample of 8,282 emergency department admissions who were administered ketamine and found no correlation between the incidence of laryngospasm with the patient’s age, dose, underlying physical illness, route, or simultaneous administration of anticholinergics and, hence, concluded that the incidents of laryngospasm were idiosyncratic and not related to the dose of ketamine used [80]. An emergency series reported that the incidence of laryngospasm was only 0.4 %. Out of the 11,589 patients studied, only two required an intervention [9,81].

Other side effects of ketamine include jerky non-purposeful movements, muscle twitching, and nystagmus. However, these are transient and do not require treatment [9]. The drug’s effect on increasing intracranial pressure is still debated, with some studies showing that it actually decreased intracranial pressure in the subjects [22]. Nausea and vomiting are some of the most typical side effects of the drug seen in the pediatric age group, especially in children older than eight years [82].

Emergence phenomenon is very uncommon in children. However, it may occur in adolescents. It is defined as an abnormal mental state developing when the subject transitions from unconsciousness induced by the general anesthetic to a complete awakening. It can present with hypoactivity or hyperactivity. A study of 745 prospective records found that only 12.5% of the children cried on awakening, 39% experienced pleasant altered sensations, and 2.1% experienced emergence delirium. Therefore, the belief that emergence delirium is a frequent side effect of ketamine is incorrect, and even a pleasant emergence phenomenon is possible [14]. These episodes can lead to anxiety on rare occassions, but it tends to resolve on their own. Premedication with midazolam has been shown to reduce the incidence of emergence reactions with ketamine sedation [83].

Emergence delirium, although common with inhalational anesthetics like sevoflurane and desflurane, is rare with ketamine. Rather, the administration of ketamine in the form of an intraoperative sedative can help prevent it [84]. Other side effects include dizziness, drowsiness, and insomnia. Psychomimetic side effects of ketamine range from unpleasant dreams to hallucinations. The incidence of these is minimized by pretreatment with propofol or by administering lamotrigine [46,85]. Urinary and liver toxicity is only seen when high doses of ketamine are given regularly. As such, the margin of safety of ketamine is high, and a single accidental overdose does not cause organ toxicity [86]. The risk of ketamine dependence is present with long-term ketamine infusions, for example, when used for chronic pain. Clinicians should be wary of this and should taper the dose of ketamine infusion when given for more than 72 hours by 5 μg/kg/min every 8-12 hours. The incidence of acute ketamine withdrawal can be treated by administering benzodiazepines to calm the patient [87].

Long-Term Complications

Ketamine is thought to be a drug of abuse, but many case reports have reported no significant tolerance or psychomimetic effects with long-term use of ketamine infusion [88]. But some research suggests that it can lead to behavioral changes like apathy/withdrawal, separation anxiety, general anxiety, insomnia, and disturbed appetite [89]. Hence, further investigation is needed to assess the long-term complications of chronic ketamine use.

## Conclusions

In summary, ketamine is a unique drug that produces dissociative anesthesia, a trance-like state characterized by catatonia, catalepsy, analgesia, and amnesia. It maintains laryngeal and pharyngeal reflexes, causes bronchodilatation, and does not lead to hemodynamic instability. Due to these invaluable advantages, it is considered the drug of choice for asthma and CHD. In recent years, several research trials and systematic reviews have suggested its efficacy as an analgesic and a sedative, especially in children. It also has several non-anesthetic uses in clinical practice, including refractory depression, PTSD, and generalized anxiety disorder. It is considered an excellent drug for acute and chronic pain, especially when it is due to a neuropathic component. However, the taboo of its side effects as well as its psychological tolerance has limited its use. There is evidence that many of these fears about its side effects are unfounded. Unfortunately, the quality of literature on the drug is still unsatisfactory. We recommend that more studies should be conducted to explore the role of ketamine in the pediatric population and determine its usefulness outside of being an anesthetic.
